# Psychometric properties of the Brief-COPE for the evaluation of coping strategies in the Chilean population

**DOI:** 10.1186/s41155-018-0102-3

**Published:** 2018-08-03

**Authors:** Felipe E. García, Carmen Gloria Barraza-Peña, Anna Wlodarczyk, Marcela Alvear-Carrasco, Alejandro Reyes-Reyes

**Affiliations:** 1 Facultad de Ciencias Sociales y Comunicaciones, Universidad Santo Tomás, Av. Prat 855, Concepción, Chile; 2 Maternal and Child Department, Faculty of Nursery, University of Concepción, Concepción, Chile; 3 Escuela de Psicología, Casa Central, Universidad Católica del Norte, Av. Angamos 0610, Antofagasta, Chile; 4 Family Health Center, Villa Nonguén, Concepción, Chile; 5 Department of Social Psychology and Methodology of Behavior Sciences, University of the Basque Country, Avenida de Tolosa 70, 20018 San Sebastián, Spain

**Keywords:** Brief-COPE, Coping, Stress, Subjective well-being, CFA

## Abstract

The Brief-COPE is an abbreviated version of the COPE (Coping Orientation to Problems Experienced) Inventory, a self-report questionnaire developed to assess a broad range of coping responses. Currently, it is one of the best validated and most frequently used measures of coping strategies. The aim of this study was to validate a culturally appropriate Chilean version of the Brief-COPE, assess its psychometric properties and construct and congruent validity. The Spanish version of the Brief-COPE was administrated in a community sample of 1847 Chilean adult (60.4% women) exposed to a variety of stressful experiences. The factorial structure of the inventory was examined by comparing four different models found in previous studies in Latin American population. The results of confirmatory factor analyses revealed, as in the original studies, a 14-factor structure of the Brief-COPE. These dimensions showed adequate internal structure and consistency. The factorial invariance comparing women and men confirmed strict invariance. Additionally, the results showed significant correlation between some Brief-COPE scales, such as denial and substance use, with perceived stress and emotional support and active coping with subjective well-being. Overall, the present work offers a valid and reliable tool for assessing coping strategies in the Chilean population.

## Background

Traditionally, coping has been defined as a special category of adaptation elicited in normal individuals by unusually taxing circumstances (Costa, Somerfield, and McCrae, 1996). The most influential model of psychological stress response is the one proposed by Lazarus and Folkman (1984). According to the authors, the process of coping was defined as constantly changing cognitive and behavioral efforts which are undertaken by an individual in order to deal with demands which are especially challenging and are probably exceeding individual capacities and/or resources (Lazarus and Folkman, 1984). According to these authors, the process of coping involves three main elements: the source of the stress (the event or stressor); cognitive appraisal (which includes evaluation of the event as being irrelevant, threatening or positive, and simultaneous assessment of available coping resources within the individuals and their environment); and coping mechanisms. Coping behaviors and strategies have been traditionally dichotomized into categories, such as problem- versus emotion-focused, functional versus dysfunctional, approach versus avoidance, engagement versus disengagement, and primary versus secondary control coping. Lazarus and Folkman’s (1984) method of categorizing coping behaviors into problem-focused or emotion-focused is the most famous and used one to study coping. Some behaviors, such as planned problem-solving, can be labeled *problem-focused coping* and referred to actions aimed to eliminate the stress factor or reduce its impact. On the other hand, behaviors such as distancing, self-controlling, accepting responsibility, escape/avoidance, and positive reappraisal can be categorized as *emotion-focused coping* which alludes to actions aimed to prevent, minimize, or reduce the emotional anguish caused by the stressful situation. Endler and Parker (1990) suggested adding a third set of strategies denominated avoidant strategies, which are focused on avoiding stressful situations by seeking the company of others or by engaging in different activities.

Coping strategies has been extensively studied; however, this research has not always produced consistent findings, among others, due to the complex nature of the relationship between stressors, coping strategies, and physical and mental health. Recent reviews and meta-analyses have concluded that coping dimensions are unstable and depend on the type of stress and sample (e.g., Campos, Iraurgui, Páez, and Velasco, 2004). For instance, numerous studies have indistinctly classified some specific strategies such as *religion*¸ *positive reframing*, *humor*, and *acceptance* as *problem-focused*, *emotion-focused*, *or* avoidant (Schnider, Elhai, and Gray, 2007).

Nevertheless, there is some agreement about the existence of a second-order dimension such as adaptive and maladaptive coping (Campos et al. 2004; Carver, Scheier, and Weintraub, 1989). Adaptive forms of coping include direct coping, if the problem can be solved, reappraisal, regulated emotional expression, and non-repressive self-control. The maladaptive dimension includes rigid dysfunctional approach coping (rumination, venting/emotional discharge, and confrontation) and rigid maladaptive avoidance, based on abandonment, social isolation, inhibition, and emotional suppression (Connor-Smith and Flachsbart, 2007). For instance, *religion* has been considered in some studies as a maladaptive strategy (Reich, Costa-Ball, and Remor, 2016), whereas in other studies appears with an adaptive value (García, Páez, Cartes, Neira, and Reyes, 2014). Park et al. (2004) pointed out that problem-focused strategies are appropriate if the stressor is controllable, whereas emotion-focused strategies are suitable if the stressor seems uncontrollable. On the other hand, avoidant strategies would allow a gradual recognition of the threat, which might also be positive in the case of uncontrollable situations (Rodríguez, Pastor, and López, 1993).

Moreover, Matud (2004) explored gender differences in stress and coping and found that, in general terms, women suffer more psychological distress than men and their coping style is more emotion-focused than that of men. Accordingly, Carver et al. (1989) found several significant gender differences in the reported use of coping strategies. That is, women showed a tendency to focus on and vent emotions, and men were using alcohol or drugs as a way of coping. In another study, on a sample of children and adolescents, it was observed that the women sought more social support and the men used more the avoidance coping strategies (Eschenbeck, Kohlmann, and Lohaus, 2007). These differences suggest that gender socialization could influence the choice of certain strategies. For example, considering that women may present a greater development in the perception of their own emotions and therefore would be more prompt to resort to them to face a stressful situation. However, it is important to consider that the decline in gender differences in socialization that has occurred in recent decades may provoke that those differences in coping would also tend to disappear (Matud, 2004).

Several scales have been designed in order to measure coping strategies (e.g., Ways of Coping Scale, Lazarus and Folkman, 1984; the Measure of Affect Regulation Styles, Larsen and Prizmic, 2006; Coping Schemas Inventory-Revised, Wong, Reker, and Peacock, 2006). One of the most popular is the COPE (Coping Orientation to Problems Experienced) Inventory (Carver et al. 1989), a multidimensional inventory that comprises 15 scales each composed of 4 items. Considering the problematic extension of the original instrument, Carver (1997) presented an abbreviated version, the Brief-COPE, which has been widely used in health contexts. This instrument has 14 subscales composed of 2 items each: (a) acceptance is accepting the reality that has happened/learning to live with it; (b) emotional support is obtaining emotional support/comfort and understanding; (c) humor is making jokes about it/making fun on the situation; (d) positive reframing is trying to see the situation from a different light, make it seem more positive/look for something good in it; (e) religion is finding comfort in religious or spiritual beliefs/praying or meditating; (f) active coping is concentrating the efforts on doing something about the situation/taking action to try to make it better; (g) instrumental support is getting help and advice from other people/trying to get advice or help from others about what to do; (h) planning is trying to come up with a strategy about what to do/thinking hard about what steps to take; (i) behavioral disengagement is giving up trying to deal with it/the attempt to cope; (j) denial is saying to myself “this is not real”/refusing to believe that it has happened; (k) self-distraction is turning to work or other activities to take my mind off things/doing something to think about it less; (l) self-blaming is criticizing myself/blaming myself for things that happened; (m) substance use is using alcohol or other drugs to feel better/to help me get through it; (n) venting is saying things to let unpleasant feelings escape/expressing negative feelings.

Carver (1997) categorizes the strategies of acceptance, emotional social support, humor, positive reframing, and religion as emotion focused. On the other hand, active coping, instrumental support, and planning are considered as problem-focused strategies. Finally, behavioral disengagement, denial, self-distraction, self-blaming, and substance use and venting are considered as dysfunctional coping strategies.

Considering that coping strategies may be classified as adaptive or maladaptive depending on different factors, there are sufficient empirical evidences that point out which are the most commonly related to emotional distress or well-being. In this vain, Meyer (2001) classified the strategies measured by Brief-COPE in maladaptive coping, which included venting, denial, substance use, behavioral disengagement, self-distraction, and self-blame, and adaptive coping, including positive reframing, planning and seeking social support, active coping, use of emotional and instrumental support, acceptance, religion, and humor. Moreover, Meyer (2001) found that maladaptive strategies have a greater relationship with mental health problems such as depression. On the other hand, adaptive strategies have a stronger relationship with psychological well-being. Accordingly, maladaptive strategies have been found to be related to perceived stress and adaptive ones to satisfaction with life (Alveal and Barraza, 2015).

A comprehensive understanding of coping may be difficult if we look only at the classic coping responses to acute stressful events. Moreover, research on adults has shown that the daily hassles are associated with physical and psychological dysfunction equally or even higher than the major life events (e.g., Compas, Wagner, Slavin, and Vannatta, 1986). It is conceivable that the reliance on only one class of events reduces the probability of obtaining significant relationships between coping efforts and both positive and negative outcomes. Therefore, in the present study, we proposed to examine coping strategies implemented by Chilean adults as responses to different stressful conditions. Additionally, studies published to date confirm that coping is a key variable in the process of reducing, minimizing, or tolerating stress (e.g., Meng and D’Arcy, 2016).

Until the date, several validation studies of the Brief-COPE have been carried out in different populations and cultural contexts. As to the Spanish language version, first Perczek et al. (2000) evaluated the convergence between the scale in English and in Spanish. Subsequently, several similar studies have been conducted in Chile, Mexico, and Uruguay (Table 1).

**Table 1:** Comparative summary of factor analytic findings using the Brief-COPE in Ibero-American population (*n* = 1847)

Source	Sample	Analysis	Factors	Observations
Alveal and Barraza (2015)	333 Chilean adults, all types of events	EFA	8 factors, 24 items	Scales of acceptance and positive reframingare excluded. The following scales converge in one single factor: (a) denial and self-blaming, (b) self-distraction and behavioral disengagement, (c) active coping and planning, (d) instrumental support and emotional support.
Ornelas et al. (2013)	203 Mexican women, breast cancer	EFA	7 factors, 17 items	Scales of active coping, positive reframing, denial, behavioral disengagement, acceptance, and one item of emotional support are excluded. The followingscales converge on one single factor: (a) instrumental support by venting, (b) religion and an item of emotional support.
Reich et al. (2016)	203 Uruguayan women, all types of events	EFA	4 factors, 24 items	Scales of self-blaming and instrumental support are excluded. The following scales converge on a single factor: (a) a self-distraction, humor, an item of positive reframing, and an itemof venting, (b) denial, behavioral disengagement, acceptance, and an item of venting, (c) substance use and religion, (d) activecoping, planning, emotional support, and an item of positive reframing.

In the Chilean context, the study by Alveal and Barraza (2015) indicated the presence of eight factors (excluding scales of acceptance and positive reframing): (a) negative coping: denial and self-blaming, (b) avoidance and resignation: self-distraction and behavioral disengagement, (c) problem-focused coping: active coping and planning, (d) social support: instrumental support and emotional support, (e) humor, (f) venting, (g) religion, and (h) use of substance. Nevertheless, considering that the authors used exploratory factor analysis, it was not possible to confirm the first-order nor the second-order factor structure.

In Mexico, Ornelas et al. (2013) performed a principal component analysis of the instrument with 203 patients with breast cancer. In this study, the authors reduced the instrument to only 17 items, since the scales of active coping, positive reframing, denial, behavioral disengagement, acceptance, and one item of emotional support were excluded. They maintained the subscales of planning, self-distraction, humor, self-blame, and use of substance. The following scales converge on one single factor: (a) instrumental support and venting, (b) religion and an item of emotional support. As in the previously mentioned Chilean study, the authors did not determine the existence of second-order factors.

Finally, in Uruguay, Reich et al. (2016) performed an exploratory factor analysis of the instrument, with 203 adult females belonging to the general population. They found that the 24 items were grouped into four factors: (a) self-distraction, humor, an item of positive reframing, and an item of venting, (b) denial, behavioral disengagement, acceptance and an item of venting, (c) substance use and religion, and (d) active coping, planning, emotional support and an item of positive reframing. The scales of self-blaming and instrumental support were excluded. In addition, they rated the different strategies in focus on the problem, on the emotion, or on the avoidance but did not carry out a second-order analysis.

As aforementioned, the existing studies present important limitations. First, they performed only exploratory factorial analysis or principal component analysis. And second, the samples were relatively small, two of those studies explored coping strategies of people affected by stressful situations (Alveal and Barraza, 2015; Reich et al. 2016), and one used a sample of women diagnosed with cancer (Ornelas et al. 2013). Hence, we consider that it is necessary to evaluate the proposed factor structures through a confirmatory factorial analysis and in a large sample in order to be able to provide a robust version of the Brief-COPE scale for its use in Latin American.

### Current study

The aim of the current study is to present psychometric properties of the Brief-COPE in Chilean adult population exposed to different stressful events. Specifically, we aimed at examining the factorial structure of this measure and evaluating the reliability and the validity of the construct. Moreover, we aim at examining measurement invariance of the scale among men and women. Furthermore, we pretend to examine the relation between coping strategies indicators of positive mental health such as the subjective wellbeing, and negative mental health such as the perceived stress. The design is a correlational and descriptive study. Data were collected in one single temporary cut, so it is a cross-sectional study.

## Methods

### Participants

Participants in the study were 1847 adults, 60.4% women and 39.6% men aged between 18 and 86 (*M* = 39.39; SD = 13.58), inhabitants of different provinces of Chile. They were selected by a non-probabilistic sampling, with a criterion of convenience, looking for people exposed to different stressful events either belonging to groups of self-help (parents of autistic children, caregivers of chronically ill), belonging to a specific labor condition (officials of urgency services), populations exposed to natural disasters (earthquake in Chile, 2010), specific clinical populations (users with headache disorders or labor accidents), or people individually recruited (death of a relative, breakup, traffic accident, assaults, etc.). They were selected after acknowledging being exposed to different stressful conditions present in a list. According to this, 26.8% was exposed to labor and/or academic stress, 15.1% was exposed to police violence, 15% suffers or suffered a serious or incapacitating illness, 9.4% was affected by a serious and/or incapacitating illness in a close relative, 8.2% was exposed to a natural disaster, 5.7% was affected by the death of a close person, 4.7% was affected by a labor or traffic accident, 4.4% suffered a breakup, and the remaining 10.7% was affected by other highly stressful events.

### Instruments

#### Coping strategies

The Brief-COPE (Carver, 1997) in a translation for Spanish population by Moran et al. (2010) was used. The instrument consists of 28 items that measure 14 factors of 2 items each, which correspond to a Likert scale ranged from 0 = I have not been doing this at all to 3 = I have been doing this a lot. The psychometric properties of the scale are presented in the “Results” section.

#### Subjective well-being

It was measured with the Satisfaction with Life (SWL) Scale developed by Diener et al. 1985 (translation by Moyano Díaz and Ramos Alvarado, 2007). The scale has five items that are answered on a Likert scale ranged from 1 (totally disagree) to 7 points (totally agree). In the present study, the scale accounted for high internal consistency (*α* = 0.85).

#### Perceived stress

It was measured using the Perceived Stress Scale (PSS-14; Cohen, Kamarck, and Mermelstein, 1983; translated and validated by Tapia, Cruz, Gallardo, and Dasso, 2007). In the previous study by Tapia et al. (2007), it was found to be positively correlated with state and trait anxiety and with major depression, as pointed out by Pedrero and Olivar (2010). The scale is composed of 14 items with a Likert response format that is answered on a scale that ranges from 0 (never) to 4 (very often). The reliability coefficient presented adequate values (*α* = 0.77).

#### Socio-demographic questionnaire

An ad hoc questionnaire was prepared, in which information about gender, age, and type of stressor is registered. For the type of stressor, a mixed question with 10 response options was used (based on Norris, Hamblen, Brown, and Schinka, 2008): natural disaster, personal serious illness, serious illness of a very close person, home accident, labor or traffic accident, death of a very close person, breakup, state violence, domestic violence, and criminal violence. To these, another open question was added (“another, please mention”).

### Procedure

A written authorization via e-mail was obtained from the author of the Brief-COPE Charles S. Carver for validating the instrument in the Chilean population. The instrument was applied in different provinces of Chile, covering 3000 km from the city of Antofagasta to the Aysen Regions. The Brief-COPE scale was applied to all participants, whereas the other scales were applied to different subsamples. Surveys were applied with paper and pencil by graduated psychology students. Informed consent was obtained from all individual participants included in the study. The study was approved by the Ethics Committee of the St. Thomas University (No. 89/2014).

### Data analysis

In order to evaluate the construct validity of the scale, we first conducted a confirmatory factorial analysis (CFA) with maximum likelihood estimation, trying to test four different models: original theoretical structure (Carver, 1997), and the exploratory factorial structures found in other studies carried out in Latin America, specifically in Chile (Alveal and Barraza, 2015), Mexico (Ornelas et al. 2013), and Uruguay (Reich et al. 2016).

In order to evaluate the fit of the model, we considered various fit indices (Hu and Bentler, 1999): (a) *χ*^2^, a non-significant value indicates a good fit; (b) *χ*^2^/*df*, a good fit is suitable for a value less than 2; confirmatory fit index (CFI) and Tucker-Lewis index (TLI), a value ≥ 0.90 indicates an acceptable fit, whereas a value ≥ 0.95 is indicative of good fit; (c) parsimony normed fit index (PNFI), values greater than 0.50 are considered suitable; (d) root mean square error of approximation (RMSEA), a value ≤ 0.05 (90% CI ≤ 0.08) is indicative of good fit; (e) Akaike’s information criterion (AIC) is a comparative indicator, whereas lower values favor the choice of model.

Considering previous findings on gender differences in stress and coping (see Matud, 2004), once the factorial structure with better fit indices has been established, the factorial invariance was performed comparing women and men through successive multi-sample CFA by comparing nested models with CFI, TLI, RMSEA, and AIC fit indices. Since the chi-squared test is sensitive to the size of the sample, and following the indications by Cheung and Rensvold (2002), we considered the decreases in CFI of less than 0.01 (Δ ± 0.01) when compared to the previous model as a more adequate indicator of invariance. In addition, a CFI and a TLI greater than 0.90 and an RMSEA less than 0.08 are expected. In the case of AIC, showing significant variation between one model and the next is not expected, so that the models are considered acceptable.

Subsequently, we calculated the descriptive statistics and the internal consistency of the Brief-COPE and its subscales. We used Cronbach’s for the evaluation of the reliability; nevertheless, considering that the use of alpha coefficient is not recommendable for the scales which are composed of two items, we present these values only as a referential information. Additionally, we calculated Pearson’s *r* correlations between the items of each of the subscales.

Following this analysis, the relation between the subscales of the Brief-COPE was established with scales of life satisfaction (*n* = 712) and perceived stress (*n* = 603), using Pearson’s *r* correlation in order to differentiate the functional strategies of the dysfunctional strategies. For all the presented statistical data analysis, we used the IBM SPSS Statistics 20 (IBM Corp., 2010) and AMOS v18 (IMB Corp. 2010).

## Results

First, we conducted a series of CFA in order to compare four previously proposed models. Model 1 corresponds to the theoretical model proposed by Carver (1997), and it is composed of 28 items and 14 factors. Model 2 corresponds to the results of exploratory factor analysis performed by Alveal and Barraza (2015) using a Chilean sample, and it is composed of 24 items and 8 factors. Model 3 corresponds to the results of the exploratory factor analysis performed on a Mexican sample by Ornelas et al. (2013), this version was composed of 17 items and the authors identified 7 factors. Finally, model 4 is based on the results of the exploratory factor analysis performed on Uruguayan sample by Reich et al. (2016); in this case, the authors used 24 items and identified 4 factors. As can be observed in Table 2, the model which obtained the best fit was model 1, which was composed of 28 items corresponding to 14 original factors of the Brief-COPE.

**Table 2:** Fit indices for the hypothesized model (*n* = 1847)

	*χ* ^2^	*df*	*χ* ^2^ */df*	CFI	NFI	TLI	PNFI	AIC	RMSEA	CI 90%
Model 1	1079.42**	259	4.17	0.94	0.92	0.91	0.59	1429.42	0.04	0.04–0.05
Model 2	1836.96**	224	8.20	0.87	0.51	0.82	0.64	2036.96	0.06	0.06–0.07
Model 3	1689.64**	98	17.33	0.79	0.78	0.68	0.50	1842.64	0.10	0.09–0.10
Model 4	5399.09**	246	21.95	0.53	0.52	0.43	0.43	5555.09	0.11	0.11–0.11

*Note*: *CFI* comparative fit index, *NFI* normed fit index, *TLI* Tucker-Lewis index, *PNFI* parsimonious normed fit index, *AIC* Akaike’s information criterion, *RMSEA* root mean square error of approximation, *CI* confidence interval

**p < 0.001

In Table 3, the factorial loadings of model 1 are presented. Here, it can be observed that all items contribute with their respective factor with loadings greater than the recommended minimum of 0.40 (Hair, Black, Babin, Anderson, and Tatham, 2005).

**Table 3:** Factors, items, and standardized factor loadings for model 1 (*n* = 1847)

Factor/items	Standardized factor loading
Instrumental support	
1. I have been trying to get advice or help from other people about what to do. [Intenté conseguir que alguien me ayudara o aconsejara sobre qué hacer.]	0.69
28. I have been getting comfort and understanding from someone. [Conseguí que otras personas me ayudaran o aconsejaran.]	0.85
Emotional Support	
9. I have been getting emotional support from others. [Conseguí apoyo emocional de otros.]	0.79
17. I have been getting comfort and understanding from someone. [Conseguí el consuelo y la comprensión de alguien.]	0.78
Active Coping	
2. I have been concentrating my efforts on doing something about the situation I’m in. [Concentre mis esfuerzos en hacer algo sobre la situación en la que estaba.]	0.60
10. I have been taking action to try to make the situation better. [Tomé medidas para intentar que la situación mejorara.]	0.80
Planning	
6. I have been trying to come up with a strategy about what to do. [Intenté proponer una estrategia sobre qué hacer.]	0.67
26. I have been thinking hard about what steps to take. [Pensé detenidamente sobre los pasos a seguir.]	0.65
Acceptance	
3. I have been accepting the reality of the fact that it has happened. [Acepté la realidad de lo que había sucedido.]	0.54
21. I have been learning to live with it. [Aprendí a vivir con ello.]	0.68
Self-distraction	
4. I have been turning to work or other activities to take my mind off things. [Recurrí al trabajo o a otras actividades para apartar las cosas de mi mente.]	0.62
22. I have been doing something to think about it less, such as going to movies, watching TV, reading, daydreaming, sleeping, or shopping. [Hice algo para pensar menos en ello, tal como ir al cine o ver la televisión].	0.68
Denial	
5. I have been saying to myself “this is not real.”. [Me dije a mí mismo “esto no es real.”]	0.69
13. I have been refusing to believe that it has happened. [Me negué a creer que había sucedido.]	0.80
Humor	
7. I have been making jokes about it. [Hice bromas sobre ello.]	0.79
19. I have been making fun of the situation. [Me reí de la situación.]	0.84
Self-blaming	
8. I have been criticizing myself. [Me critiqué a mí mismo.]	0.70
27. I have been blaming myself for things that happened. [Me eché la culpa de lo que había sucedido.]	0.64
Behavioral disengagement	
11. I have been giving up trying to deal with it. [Renuncié a intentar ocuparme de ello.]	0.61
25. I have been giving up the attempt to cope. [Renuncié al intento de hacer frente al problema.]	0.60
Venting	
12. I have been saying things to let my unpleasant feelings escape. [Dije cosas para dar rienda suelta a mis sentimientos desagradables.]	0.78
23. I have been expressing my negative feelings. [Expresé mis sentimientos negativos.]	0.66
Positive reframing	
14. I have been trying to see it in a different light, to make it seem more positive. [Intenté verlo con otros ojos, para hacer que pareciera más positivo.]	0.56
18. I have been looking for something good in what is happening. [Busqué algo bueno en lo que estaba sucediendo.]	0.76
Substance use	
15. I have been using alcohol or other drugs to make myself feel better. [Utilicé alcohol u otras drogas para hacerme sentir mejor.]	0.85
24. I have been using alcohol or other drugs to help me get through it. [Utilicé alcohol u otras drogas para ayudarme a superarlo.]	0.82
Religion	
16. I have been trying to find comfort in my religion or spiritual beliefs. [Intenté hallar consuelo en mi religión o creencias espirituales.]	0.81
20. I have been praying or meditating. [Recé o medité.]	0.82

Once the factorial structure of fourth dimensions in the Brief-COPE was confirmed, it was proceeded to assess the invariance of the model in the two samples: women and men. For this, the sequential evaluation of the configural, metric, strong, and strict invariance was performed (Elosua, 2005). The configural invariance is the basic model of analysis in any study of equivalence, and it is required that factors must be specified for the same items in both populations. The rejection of the hypothesis of configural invariance implies the lack of substantial equivalence of constructs among populations. The metric invariance examines the equality of regression coefficients. The strong invariance examines the equality in the intercepts. Finally, the strict invariance examines the equality in the variance and covariance of errors and is the highest attainable standard of accord among factorial structures.

The analysis between the two groups showed the existence of configural invariance, since the values of RMSEA, TLI, and CFI fit indices were acceptable. It also shows metric invariance, since the CFI reduced its value by 0.01 over the previous model and other indices nearly suffered no variations. Thus, it can be concluded that the factor loadings are equivalent in the two subsamples. The strong invariance is also acceptable since the fit indices continue to be appropriate and the CFI reduces in a 0.01, so it is possible to conclude that the two evaluated models are equivalent with respect to the factorial coefficients and the intercepts. Finally, the strict invariance also shows a decrease of 0.01 in the CFI and acceptable fit values in the different indices, so it is also accepted. This has reached the maximum level of invariance to which the model has been tested (see Table 4).

**Table 4:** Factorial invariance models between women (*n* = 1115) and men (*n* = 732)

Models	*χ* ^2^ *(df)*	Δ*χ*^2^	CFI	ΔCFI	TLI	RMSEA	AIC
M1: configural	1530.59 (518)	–	0.93	–	0.89	0.03	2230.59
M2: metric	1550.46 (532)	− 19.87	0.93	− 0.01	0.89	0.03	2222.46
M3: strong	1715.15 (560)	− 164.69	0.92	− 0.01	0.89	0.03	2331.15
M4: strict	1755.62 (588)	− 40.47	0.92	− 0.00	0.89	0.03	2315.62

*M1* = not constrained; *M2* = M1 + invariant factor loadings; *M3* = M2 + invariant intercepts; *M4* = M3 + invariant error variances and covariances

*Note*: *CFI* comparative fit index, *TLI* Tucker-Lewis index, *RMSEA* root mean square error of approximation, *AIC* Akaike’s information criterion

Descriptive data of each Brief-COPE subscale are shown in Table 5. When comparing strategies by gender, we observe that women scored higher than men on instrumental support, emotional support, self-distraction, denial, behavioral disengagement, venting, and religion. On the other hand, despite the internal consistency analysis is not recommended for subscales formed by two items, Cronbach’s alpha value is included as a reference for the reader. The results of the correlation between the two items that form each subscale are presented as supplementary data. Cronbach’s alpha for the total scale is adequate, because all values exceed the minimum value of 0.60 suggested by Nunnally and Bernstein (1995) for research purposes.

**Table 5:** Descriptive statistics for total group (*n* = 1847) and for gender (female = 115; male = 732), Cronbach’s alpha, and Pearson’s *r* correlation

	Total group		Female		Male		*t* value	*α*	*r*
	M	SD	M	SD	M	SD			
Instrumental support	2.44	1.66	2.55	1.66	2.28	1.65	3.350***	0.73	0.54***
Emotional support	2.84	1.79	3.01	1.78	2.60	1.77	4.901***	0.75	0.60***
Active coping	3.80	1.55	3.82	1.52	3.76	1.59	0.824	0.65	0.48***
Planning	3.43	1.62	3.41	1.58	3.47	1.68	− 0.887	0.62	0.45***
Acceptance	3.93	1.44	3.90	1.46	3.96	1.42	− 0.933	0.53	0.37***
Self-distraction	2.88	1.69	3.02	1.66	2.66	1.71	4.407***	0.58	0.40***
Denial	1.33	1.58	1.50	1.64	1.08	1.43	5.777***	0.71	0.55***
Humor	2.00	1.80	1.95	1.79	2.09	1.81	− 1.662	0.80	0.67***
Self-blame	2.07	1.61	2.05	1.65	2.10	1.55	− 0.601	0.61	0.44***
Behavioral disengagement	1.09	1.34	1.14	1.37	1.02	1.30	2.021*	0.54	0.37***
Venting	2.04	1.60	2.20	1.60	1.80	1.58	5.275***	0.66	0.50***
Positive reframing	3.25	1.62	3.27	1.54	3.20	1.72	0.897	0.61	0.44***
Substance use	0.64	1.29	0.60	1.24	0.71	1.36	− 1.690	0.82	0.70***
Religion	2.83	2.01	3.12	1.97	2.40	1.99	7.611***	0.79	0.65***

*p < .05; ***p < .001

By evaluating the bivariate relations between the 14 factors of model 1 (Table 6), it can be noted that the higher coefficients are observed between instrumental support and emotional support (*r* = 0.65) and between active coping and planning (0.56). This justifies their clustering in factors of four items, as proposed by Moran et al. (2010).

**Table 6:** Correlation matrix for the 14 coping dimensions model 1 (*n* = 1847)

	2	3	4	5	6	7	8	9	10	11	12	13	14
1	0.65**	0.40**	0.30**	0.13**	0.36**	0.18**	0.09**	0.13**	0.11**	0.16**	0.27**	0.13**	0.26**
2	–	0.33**	0.19**	0.20**	0.35**	0.16**	0.09**	0.07*	0.08**	0.20**	0.24**	0.08**	0.32**
3		–	0.56**	0.32**	0.21**	− 0.04	0.14**	0.13**	− 0.07*	0.04	0.37**	0.00	0.19**
4			–	0.27**	0.21**	− 0.05	0.16**	0.17**	− 0.05	0.05	0.44**	0.05	0.23**
5				–	0.16**	− 0.14**	0.12**	− 0.07*	− 0.04	0.01	0.31**	− 0.02	0.19**
6					–	0.15**	0.15**	0.15**	0.25**	0.19**	0.25**	0.14**	0.21**
7						–	0.01	0.24**	0.36**	0.26**	− 0.02	0.16**	0.09**
8							–	0.22**	0.09**	0.18**	0.22**	0.10**	− 0.05
9								–	0.18**	0.23**	0.11**	0.16**	0.02
10									–	0.23**	0.00	0.16**	0.07*
11										–	0.00	0.13**	0.06*
12											–	0.00	0.26**
13												–	0.05
14													–

*1* instrumental support, *2* emotional support, *3* active coping, *4* planning, *5* acceptance, *6* self-distraction, *7* denial, *8* humor, *9* self-blaming, *10* behavioral disengagement, *11* venting, *12* positive reframing, *13* substance use, *14* religion

*p < .01; **p < .001

The relation between different coping strategies with subjective well-being and perceived stress as indicators of mental health positive and negative respectively can be observed in Table 7. Thus, both adaptive and maladaptive strategies can be determined. It is clear that there are clearly strategies such as emotional support, active coping, planning, acceptance, and positive reframing, as well as the grouped factors of problem-focused coping and social support. In turn, clearly maladaptive strategies such as self-distraction, denial, self-blaming, behavioral disengagement, and substance use can be also observed. Other strategies seem to be more ambiguous, such as instrumental support and religion, which are positively related to both perceived stress and subjective well-being. Humor has a negative relation with perceived stress but shows no significant relation with well-being. Finally, venting presents a significant relation with well-being, but not with stress.

**Table 7:** Correlations of coping strategies with subjective well-being and perceived stress

Coping strategies	Subjective well-being (*n* = 815)	Perceived stress (*n* = 589)
Instrumental support	0.14**	0.13**
Emotional support	0.22**	0.02
Active coping	0.23**	− 0.11*
Planning	0.14**	− 0.06
Acceptance	0.11**	− 0.23**
Self-distraction	− 0.09	0.14**
Denial	− 0.09	0.28**
Humor	− 0.03	− 0.11*
Self-blaming	− 0.15**	0.18**
Behavioral disengagement	− 0.18**	0.20**
Venting	− 0.13**	0.06
Positive reframing	0.12**	− 0.10
Substance use	− 0.10*	0.20**
Religion	0.12**	0.17**

*p < 0.01; **p < 0.001

## Discussion

The main objective of this study was to establish the construct validity of the Brief-COPE. In order to verify which was the most adequate solution, we compared different models previously proposed in studies with Latin American samples. We found that the 14-factor model corresponding to the original structure of the Brief-COPE was the one which obtained the best fit.

In addition, we found the support for the strict factorial invariance in the compared groups of men and women. This means that the gender differences found in this and other studies are not due to an instrument bias, but rather to real differences in preferences expressed by women and men, since the scale has a similar structure in both. Those results are congruent with those presented by Doron et al. (2014).

By observing the internal consistencies of the different subscales, it is observed that the most of them exceed the value of 0.60 (Nunnally and Bernstein, 1995). Although Cronbach’s alpha is not recommended when there are only two items, the low internal consistency of the acceptance is reiterated in several studies. For this reason, it is recommended to exclude it from the analyses when its alpha value is less than 0.60.

Bivariate correlation between different coping strategies shows high correlation between emotional support—instrumental support and active coping—and planning. This reinforces the notion that these strategies could be measured as a factor of social support and other problem-focused coping.

We performed bivariate correlations with subjective well-being and perceived stress with the aim of distinguishing functional from dysfunctional strategies. This distinction deserves a separate discussion, mostly due to the fact that the strategies are functional or dysfunctional depending on a number of factors including the context and the elapsed time (Rodríguez et al. 1993). Thus, a specific strategy can be adaptive under certain conditions and maladaptive in others. For instance, if in illnesses such as asthma, diabetes, and cancer, which require monitoring and/or self-care behaviors for an appropriate diagnosis or treatment, being focused on the problem would result on a more effective strategy. On the contrary, in other illnesses such as palsy, being focused on the problem produces no advantages, whereas acceptance or self-distraction can be useful to reduce anxiety and depression (Rodríguez et al., 1993).

Bypassing this discussion, in the present study, it can be observed that active coping and acceptance have a positive relation with well-being and negative with stress. Therefore, in general terms, they can be considered as adaptive or functional strategies. Those results are consistent with what has been found in several studies that pointed out that active coping and acceptance could be conceived as adaptive coping (Meyer, 2001; Urcuyo, Boyers, Carver, and Antoni, 2005; Yi-Frazier et al. 2010). Thus, we can highlight their differential contribution to well-being of both of those strategies: the acceptance of negative circumstances that are not possible to be modified and active coping to resolve situations that can be modified.

On the other hand, self-blaming, behavioral disengagement, and substance use have a negative relation with well-being and positive with stress, so that they can be considered as maladaptive or dysfunctional. These three strategies together with denial have been considered in other studies as being part of a second-order coping strategy of avoidance (Doron et al. 2014).

Regarding self-blaming, despite that a direct relation with indicators of discomfort such as posttraumatic stress (Drury and Williams, 2012) or depression has been shown, this strategy received less scientific attention than other functional strategies such as substance use (Moscardino, Scrimin, Capello, and Altoè, 2014). Self-blaming implies a sense of responsibility for negative results, and it presents an attribution towards the stressful event of internal type (“I was not careful enough”) and stable (“I am a reckless person”) and, therefore, is difficult to change (Ullman, Peter-Hagene, and Relyea, 2014). On the other hand, behavioral disengagement has been described as an avoidant strategy that emerges when people expect a bad result (Carver et al., 1989). Probably, for this reason, its relationship with well-being and stress becomes so clear to these strategies.

Regarding other associations that are worth special attention, we have found a positive relationship between religion and both subjective well-being and perceived stress. This may be due to the fact that religiosity, as a coping strategy, may imply spiritual support and be related to the enhanced belief that the life is meaningful, but on the other hand, religiosity may also associated with feelings of guilt and lead to negative religious confrontations (Pargament, Smith, Koenig, and Perez, 1998). Unfortunately, this distinction is not explicitly covered by the items that compose the Brief-COPE. A similar positive relationship was found between instrumental social support and both well-being and stress. In this regard, Cohen and Wills (1985) have suggested that social support may be beneficial for people only if they are under stress conditions. In other words, coping strategies like instrumental social support would require of the presence of stress so that they may enhance well-being, and that would explain the positive relation between both.

This study has the strength of including a broad number of people exposed to different stressors and who inhabit different geographic areas of the country. However, it is not out of limitations such as the absence of other coping measures in order to estimate criterion validity, a cross-section design and retrospective that prevents us from concluding the relation between variables in terms of causality and a selection of the intentioned participants that ensures no representativeness of the sample, despite its size and variety.

Future research should intend to replicate the results of our study in different populations and cultural settings. Moreover, it is recommendable to perform probabilistic sampling within a more defined group of participants with a particular problem. Additionally, it would be convenient to introduce other variables with which could be associated with different coping strategies, such as quality of life, depression, posttraumatic stress, and posttraumatic growth. In practice, establishing which strategies that are used to cope with stress and that allow achieving positive or negative mental health can guide preventive and/or therapeutic work. For example, acceptance or active coping should be suggested as adequate coping strategies depending on whether there is or there is not a possibility to control the nature of the stressor.

## Conclusions

Overall, the present work offers researchers and professionals interested in this area of study a valid and reliable tool for assessing coping strategies in a Chilean population exposed to different types of stressors and to evaluate functionality or dysfunctionality of the use of certain strategies in such contexts. Additionally, this study provides some practical implications by pointing out that there are no important gender differences in the ways of coping with stressful life circumstances.

## Abbreviations

AIC: Akaike’s information criterion
CFA: Confirmatory factorial analysis
CFI: Confirmatory fit index
COPE: Coping Orientation to Problems Experienced
PNFI: Parsimony normed fit index
PSS: Perceived Stress Scale
RMSEA: Root mean square error of approximation
SWL: Satisfaction with Life Scale
TLI: Tucker-Lewis index
